# Supplementary material for: Semiochemicals produced by fungal bark beetle symbiont Endoconidiophora rufipennis and the discovery of an anti-attractant for Ips typographus
Source: PLoS One. 2023 Apr 6;18(4):e0283906. doi: 10.1371/journal.pone.0283906 (PMC10079057; doi:10.1371/journal.pone.0283906)
Supplement: S1 Table — (DOCX) [file pone.0283906.s001.docx]

## S1 Table: Fungal isolate used in this study

| Fungi | Isolate⃰ | Collected | Reported vector | Associated beetle | Host |
| --- | --- | --- | --- | --- | --- |
| *Endoconidiophora rufipennis* (M. J. Wingf., T.C. Harr & H. Solheim) Z. W. de Beer, T.A. Duong & M. J. Wingf  Mycobank # 810317 | **1992-633/280/7** | 15/10/1992, British Columbia, Caribou Creek, East Kootenays BC, Spillimacheen River Drain, Canada | *Dendroctonus rufipennis North American bark beetle* | *Dendroctonus rufipennis* | *Piceae engelmannii* |

⃰ The isolate was received from Dr. Paal Krokene, Norwegian Institute of Bioeconomy Research, Norway.
