# Supplementary material for: Semiochemicals produced by fungal bark beetle symbiont Endoconidiophora rufipennis and the discovery of an anti-attractant for Ips typographus
Source: PLoS One. 2023 Apr 6;18(4):e0283906. doi: 10.1371/journal.pone.0283906 (PMC10079057; doi:10.1371/journal.pone.0283906)
Supplement: S2 Table — (DOCX) [file pone.0283906.s002.docx]

## S4 Table: GLM ANOVA Tests of Between-Subjects Effects type III sum of squares for absolute catches.

| Source | SS type III | df | F | *p* (%) ^†^ |
| --- | --- | --- | --- | --- |
| Corrected Model | 2.39 | 11 | 2.3 | 3.0 * |
| (Intercept) | 402.6 | 1 | 402.6 | <0.1 *** |
| Treatment | 0.78 | 3 | 2.7 | 5.8 NS |
| Block (Area) | 1.2 | 1 | 0.4 | 0.4 ** |
| Treatment × Block | 0.4 | 6 | 0.7 | 64 NS |

Model fits: for raw catches (*x*) and for the common transformation log10(*x*+1) respectively: Normality test by Kolmogorov-Smirnov *p=* 20% and 7.9%, Shapiro-Wilk *p=* 0.7% and 8.3%, Heteroskedasticity test by Levene (median based) *p=* 18% and 10%, Breusch-Pagan for *p=* 32% and 75% (SPSS 28 UNIANOVA). Thus, better fit to assumption of normality additive variance for the log-transformed data, for which results are displayed in table body.

†) Hypothesis test indicators: NS Non-significant (p >0.05), ** p <0.0001, *** p <0.00001
