# Supplementary material for: Semiochemicals produced by fungal bark beetle symbiont Endoconidiophora rufipennis and the discovery of an anti-attractant for Ips typographus
Source: PLoS One. 2023 Apr 6;18(4):e0283906. doi: 10.1371/journal.pone.0283906 (PMC10079057; doi:10.1371/journal.pone.0283906)
Supplement: S1 File — (DOCX) [file pone.0283906.s003.docx]

# S2 Experimental procedure Synthesis and enzymatic resolution of sulcatol (3)

**Fig 1.** Synthesis and enzymatic resolution of sulcatol (3)

### Step I. Synthesis of 6-methyl-5-hepten-2-ol (3)

6-methyl-5-hepten-2-one (sulcatone, 200 mg, 1.58 mmol) was dissolved in methanol (5 mL) and sodium borohydride (0.3 eq) was added in fractions at 0 °C. Reaction mixture was allowed to reach ambient temperature and was stirred overnight. After completion of the reaction, solvent was removed under reduced pressure. Aqueous ammonium chloride (~5 mL) was added and the product was extracted with ethyl acetate (20 mL). Drying (MgSO_4_) and concentration yielded compound **3** (200 mg, 1.56 mmol, yield 99%).

**Compound 2:** GC-MS: R_t_ 20.979 min; *m/z* 43 (100%), 108, 69, 41, 55, 111, 93, 67, 126 (M^+^). In order of decreasing intensity.

**Compound** **3**: GC-MS: R_t_ 24.550 min, 25.037 min; *m/z* 95 (100%), 69, 41, 67, 110, 55, 128 (M^+^). In order of decreasing intensity.

### Step II. Enantiomeric acetylation of compound 3 using *Candida antarctica* lipase.

*Racemic*-sulcatol **3** (100 mg, 0.78 mmol) was dissolved in diisopropylether (1 mL) and 5 eq. vinyl acetate and 15 mg *Candida antarctica* lipase was added. The reaction mixture was stirred overnight at ambient temperature, then filtered and purified on silica gel chromatography to obtain compound (***S)*-3** (yield 47 mg, 0.37 mmol, yield 47%) and (*R)*-**4** (yield 65 mg, 0.38 mmol, yield 49%**).**

**Compound (*S*-3):** ^1^H NMR (500 MHz, CDCl_3_, δ in ppm) 5.15 – 5.05 (m, 1H), 3.77 (heptet, *J* = 6.1 Hz, 1H), 2.03 (dh, *J* = 14.7, 7.3 Hz, 2H), 1.86 (s, 1H), 1.66 (d, *J* = 1.9 Hz, 3H), 1.59 (s, 3H), 1.52 – 1.41 (m, 2H), 1.16 (d, *J* = 6.3 Hz, 3H). ^13^C NMR (125 MHz, CDCl_3_, δ in ppm) 131.9, 124.0, 67.8, 39.1, 25.6, 24.4, 23.3, 17.5. GC-MS: R_t_ 24.550; *m/z* 95 (100%), 69, 41, 67, 110, 71, 55, 128 (M^+^). In order of decreasing intensity. [α]_D_ = + 12.4° (*c* 0.5, DCM).

**Compound (*R*-4):** ^1^H NMR (500 MHz, CDCl_3_, δ in ppm) 5.05 (dddd, *J* = 8.6, 5.7, 2.8, 1.5 Hz, 1H), 4.90 – 4.81 (m, 1H), 2.00 (s, 3H), 1.99 – 1.94 (m, 2H), 1.65 (s, 3H), 1.63 – 1.57 (m, 1H), 1.56 (s, 3H), 1.51 – 1.42 (m, 1H), 1.18 (d, *J* = 6.3 Hz, 3H). ^13^C NMR (125 MHz, CDCl_3_, δ in ppm) 170.6, 132.0, 123.4, 70.5, 35.0, 25.6, 22.8, 21.3, 19.1, 17.5. GC-MS: R_t_ 27.114; *m/z* 95 (100%), 110, 43, 67, 69, 81, 55, 155. In order of decreasing intensity. [α]_D_ = - 2.4° (*c* 1.0, DCM).

### Step III. Acetylation of compound 3.

To a solution of compound **3** (100 mg, 0.78 mmol) in DCM (5 mL) was added trimethylamine (1 eq), DMAP (0.1 eq) and acetic anhydride (1 eq). The reaction mixture was stirred overnight at ambient temperature. The reaction mixture was diluted with aqueous ammonium chloride and extracted with DCM, washed with brine and dried over magnesium sulfate. The solvent were removed on rotavapor to afford a racemic mixture of compound **4** (131 mg, 0.77 mmol, yield 98%).

**Compound (*S*-4):** GC-MS: R_t_ 25.699 min; *m/z* 95 (100%), 110, 43, 67, 69, 81, 55, 155. In order of decreasing intensity.

**Compound (*R*-4):** GC-MS: R_t_ 27.144 min; Same fragments as for the *S*-enantiomer.

## Table 1: Sulcatone derivatives and their retention order in enantioselective GC.

| Compound | Retention time (min) |
| --- | --- |
| Sulcatone (**2**) | 20.90 |
| (*S*)-Sulcatol (*S*-**3**) | 24.60 |
| (*R*)-Sulcatol (*R*-**3**) | 25.04 |
| (*S*)-Sulcatol acetate (*S*-**4**) | 25.70 |
| (*R*)-Sulcatol acetate (*R*-**4**) | 27.14 |


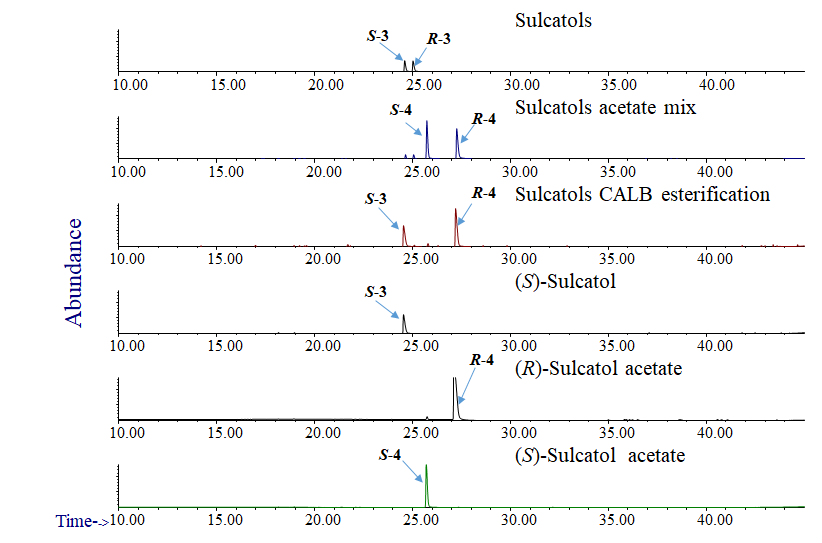


**Fig 2.** GC chromatograms of sulcatol (3) and its derivatives analysed with enantioselective Cyclosil B column.


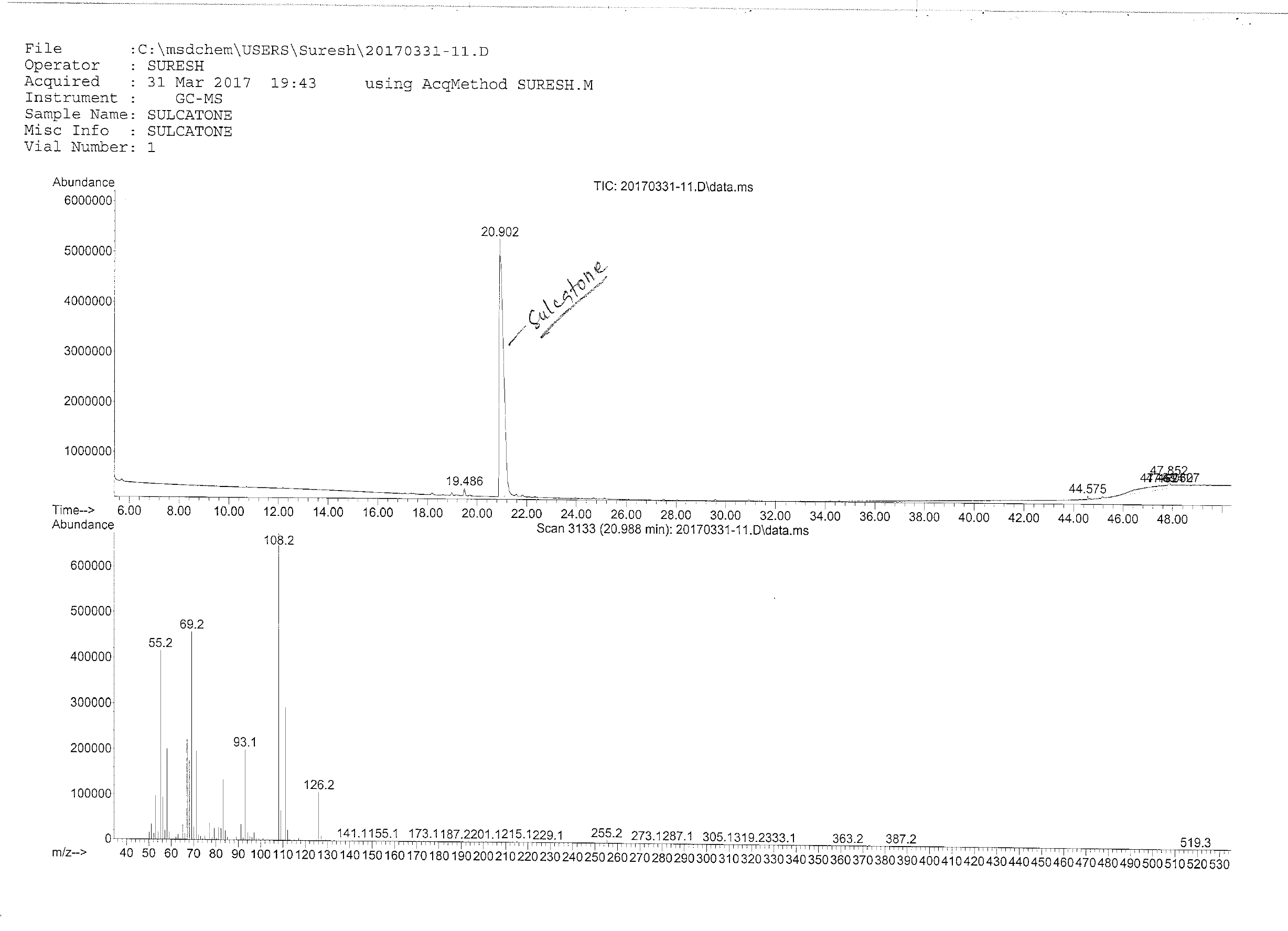


**Fig 3.** GC-MS chromatogram of sulcatone (**2**) on Cyclosil B column


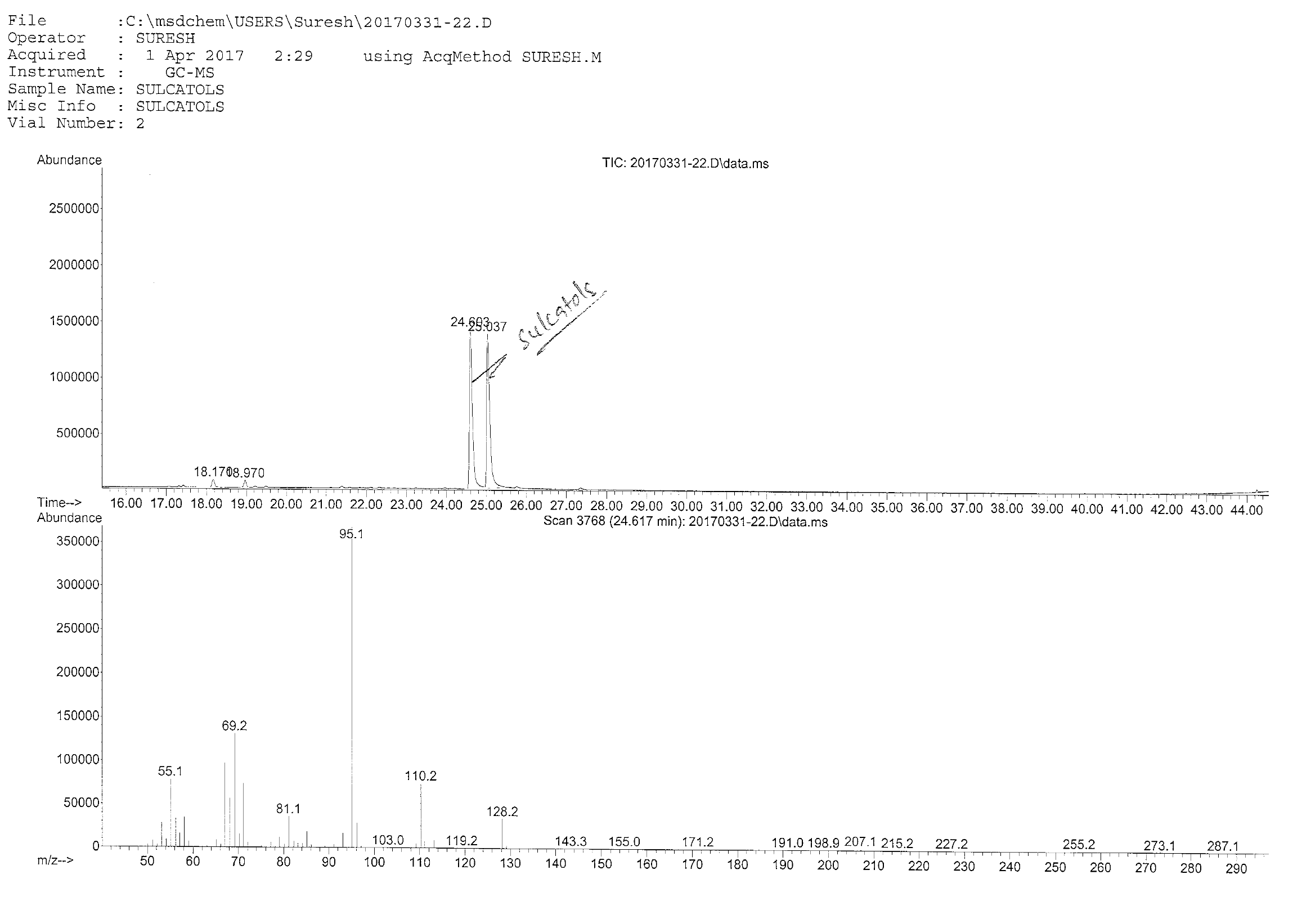


**Fig 4.** GC-MS chromatogram of *racemic-*sulcatol (**3**) on Cyclosil B column


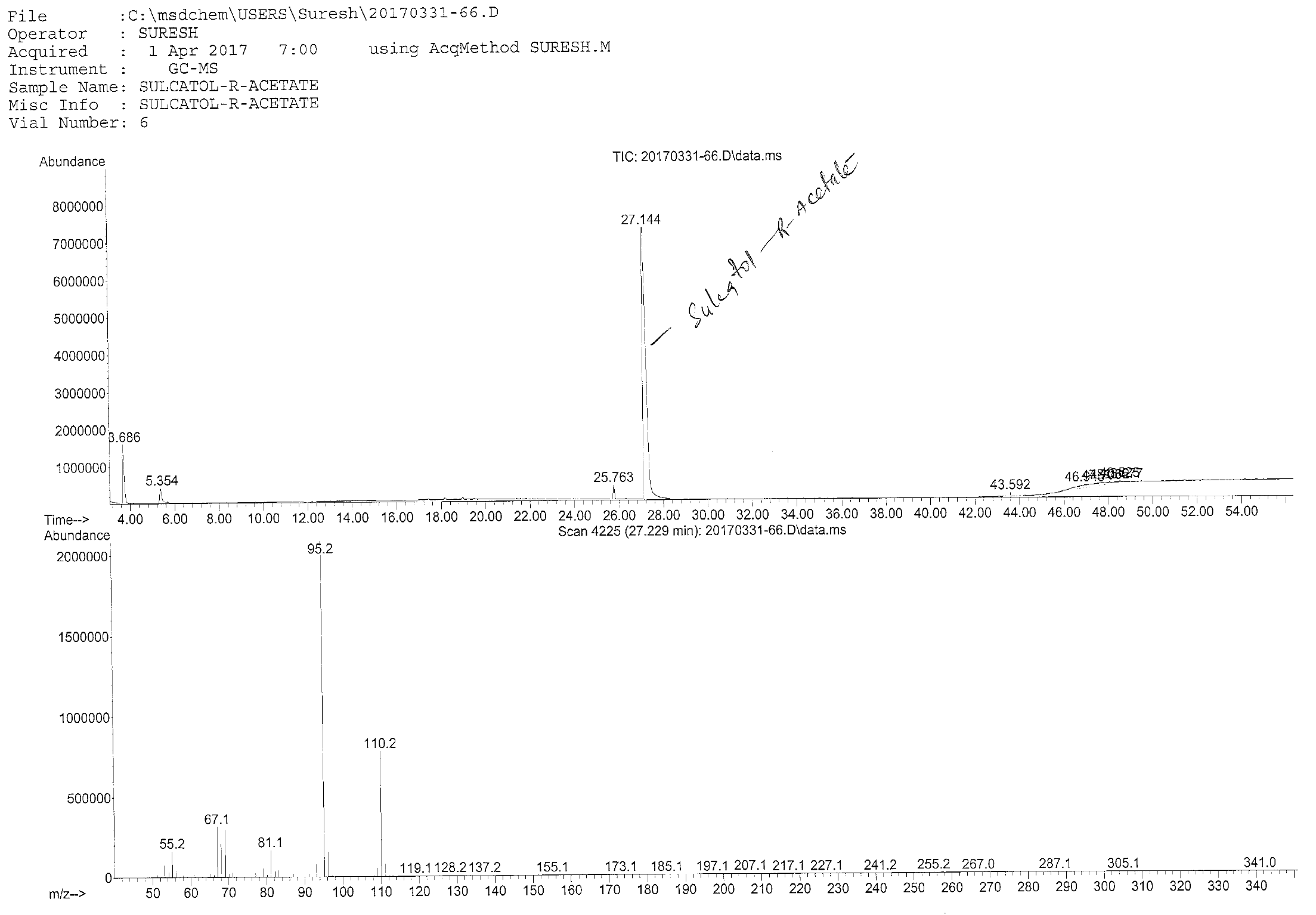


**Fig 5.** GC-MS chromatogram of (*R*)-sulcatol acetate (***R*-4**) on Cyclosil B column


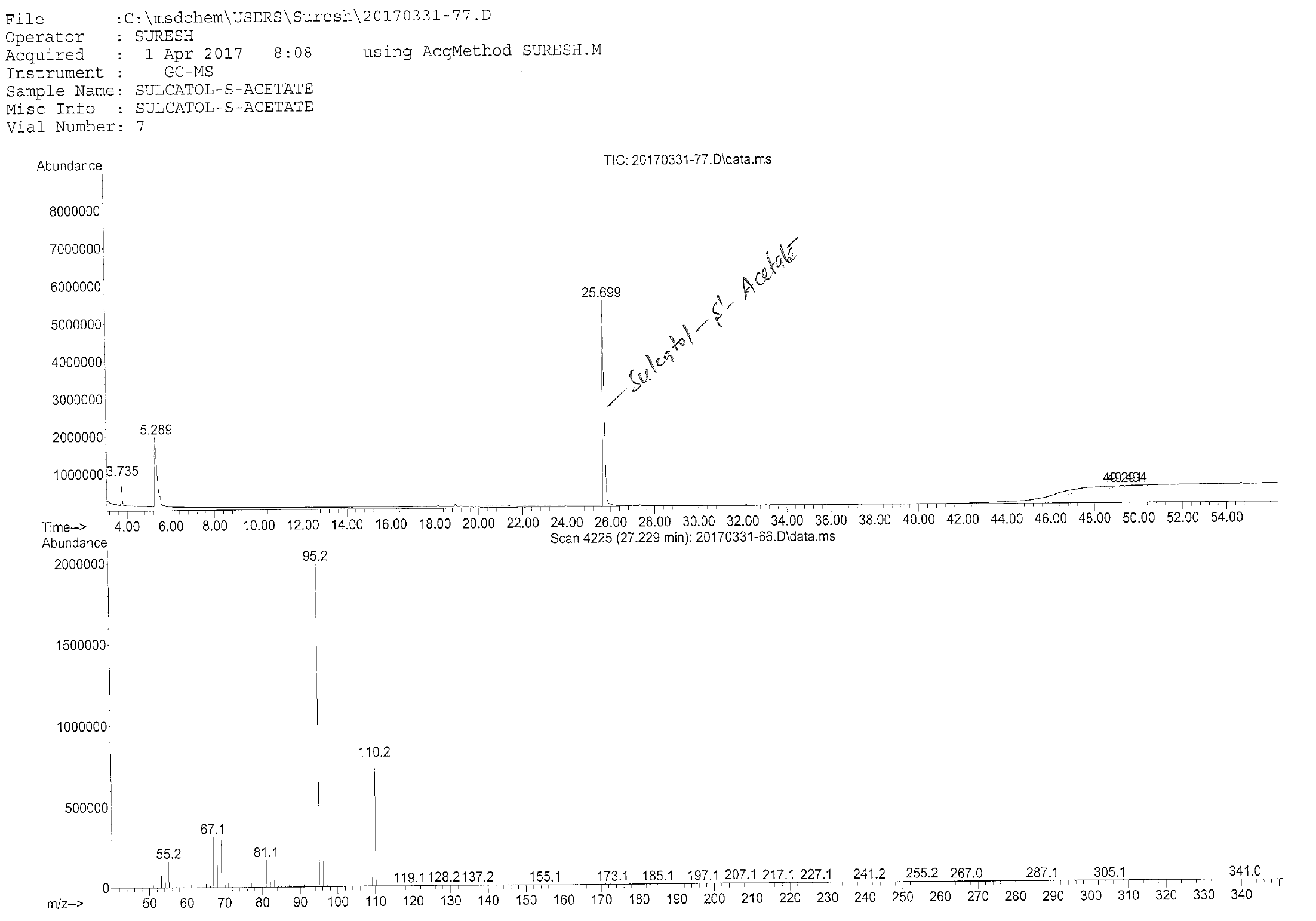


**Fig 6.** GC-MS chromatogram of (*S*)-sulcatol acetate (***S*-4**) on Cyclosil B column.


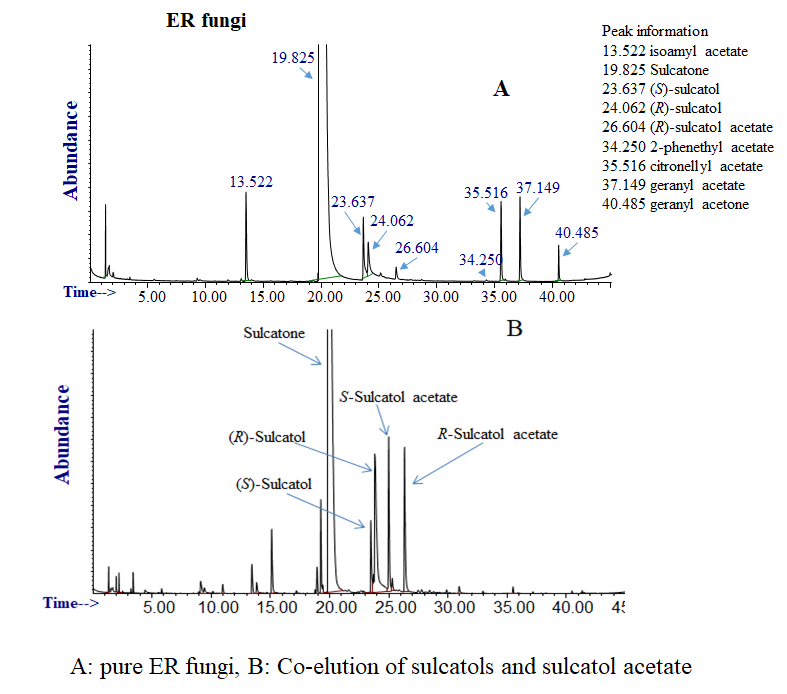


**Fig 7.** GC analysis of ER fungus volatiles and ER fungus volatiles spiked with a mixture of standard sulcatol and sulcatol acetates, analyzed on Cyclosil B column


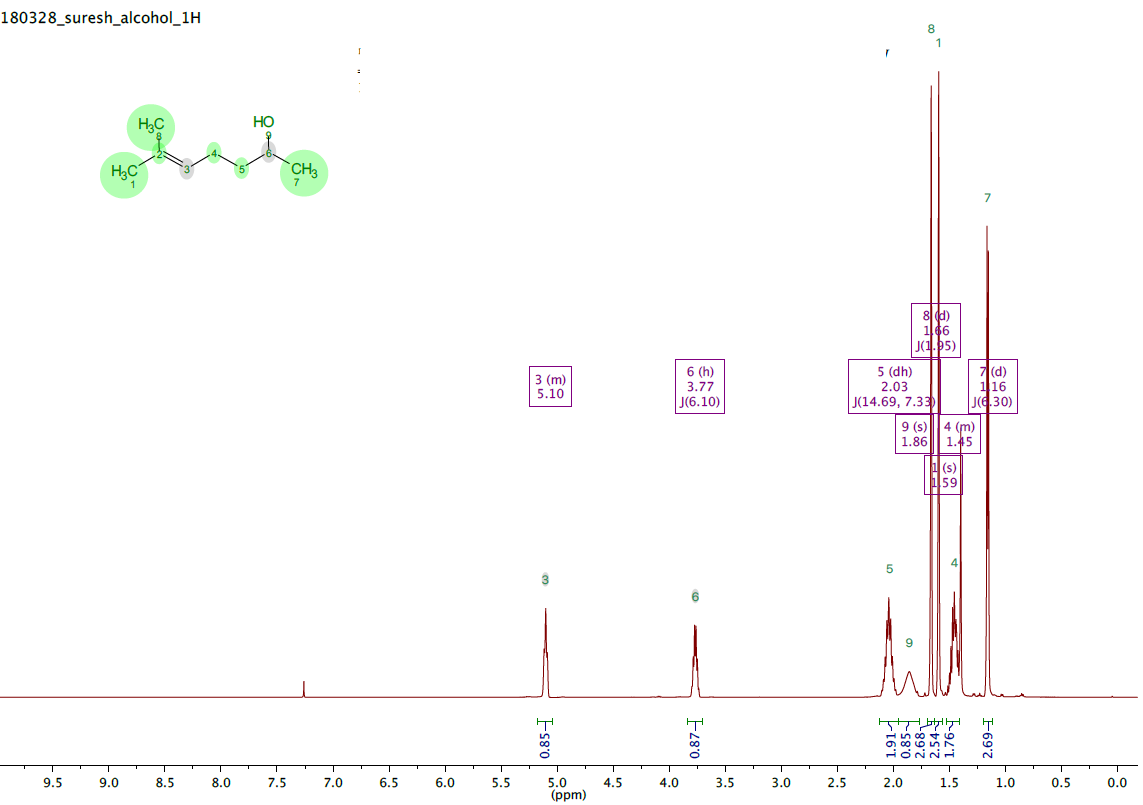


**Fig 8.** ^1^H-NMR spectrum of (*S*)*-*sulcatol (***S*-3**)


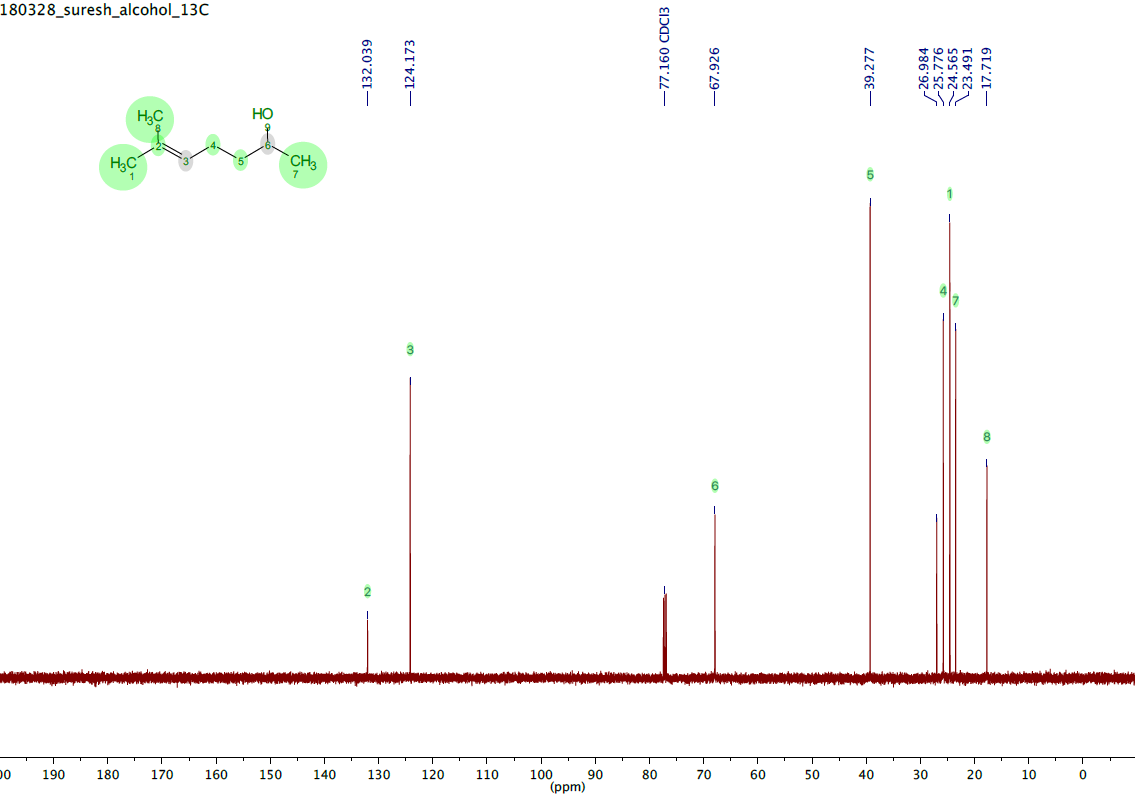


**Fig 9.** ^13^C-NMR spectrum of (*S*)*-*sulcatol (*S*-**3**)


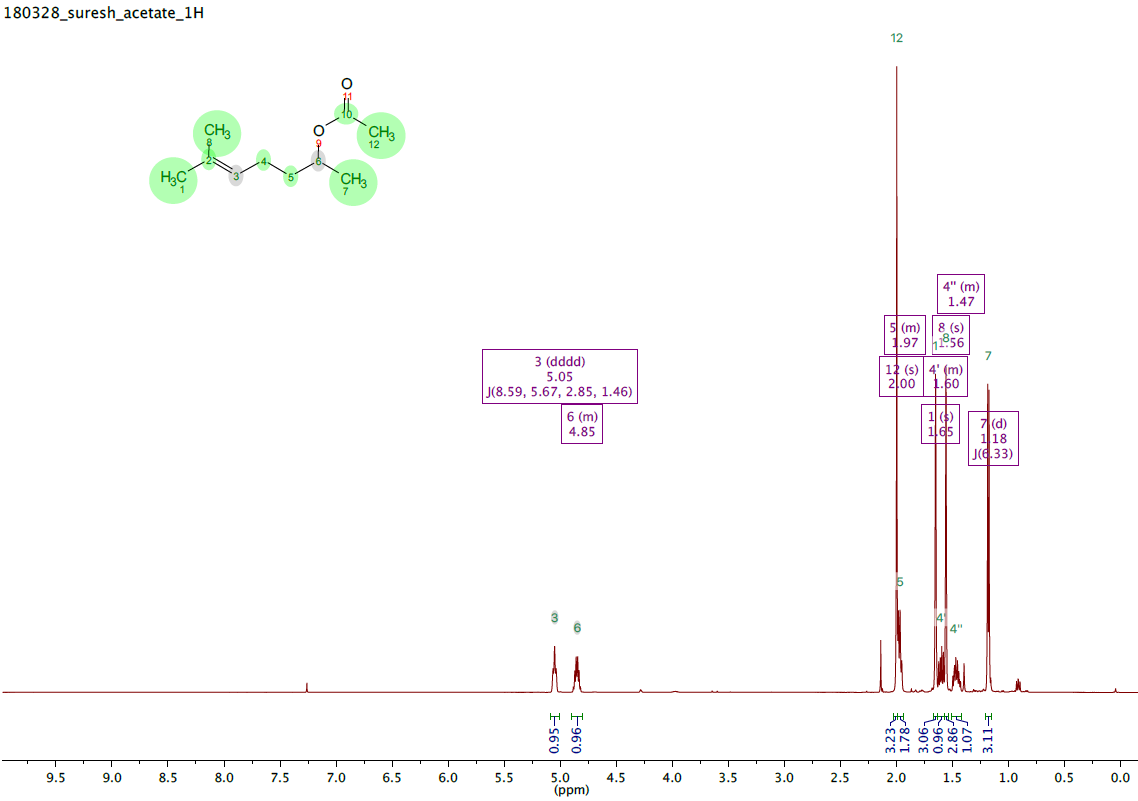


**Fig 10.** ^1^H-NMR spectrum of (*R*)-sulcatol acetate (*R*-**4**)


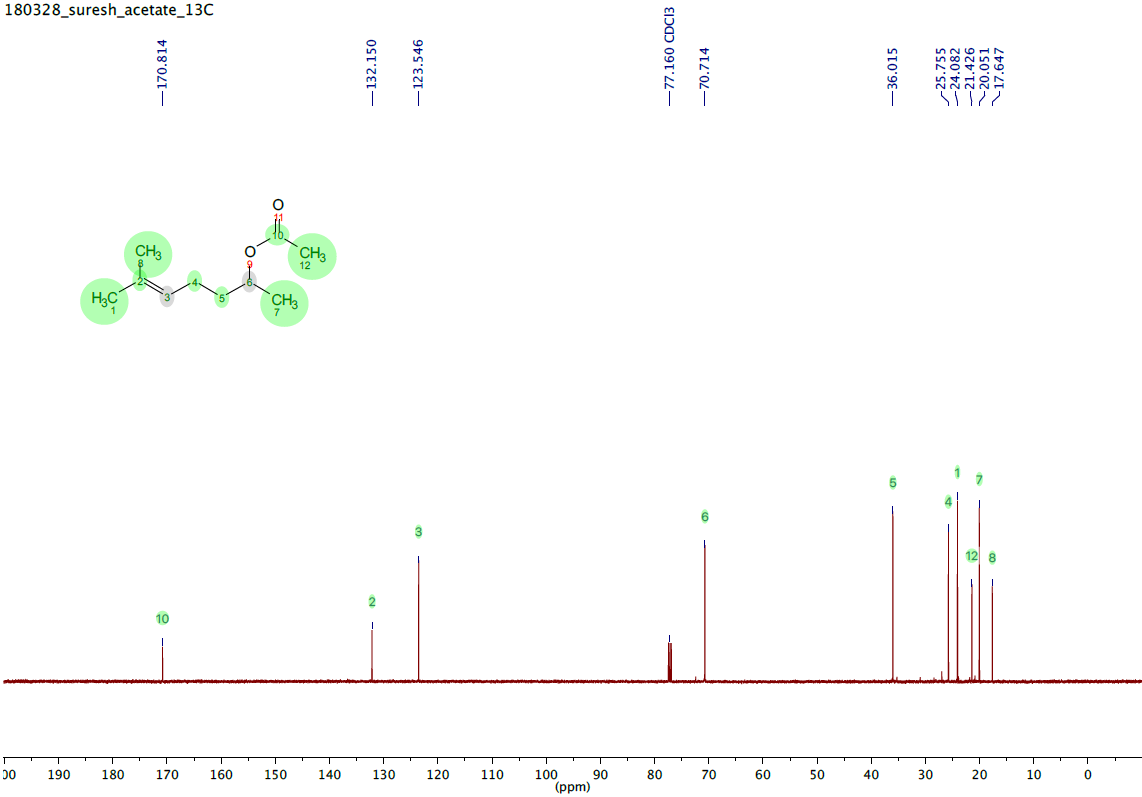


**Fig 11.** ^13^C-NMR spectrum of (*R*)-sulcatol acetate (*R*-**4**)

**
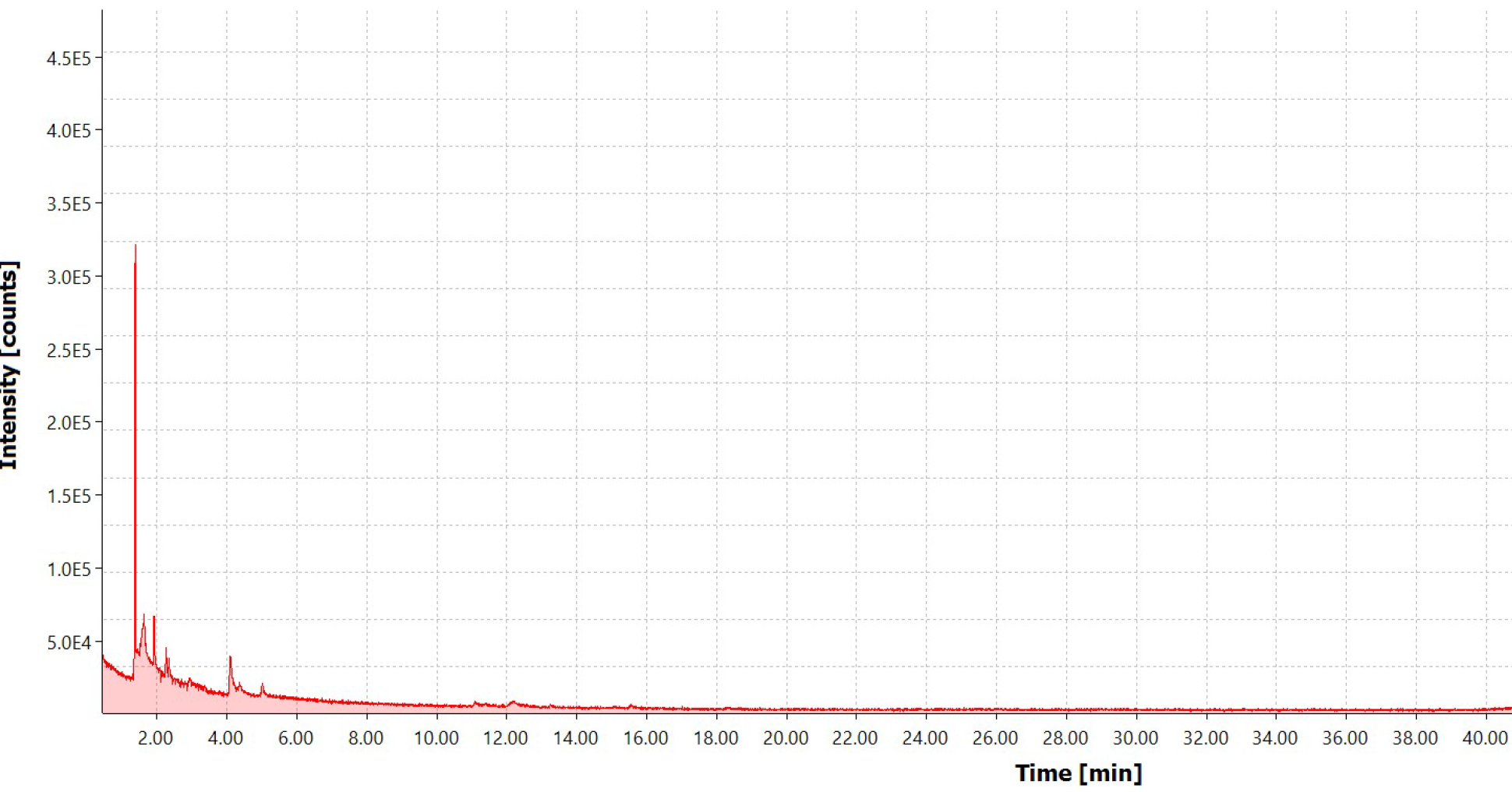
**

**Fig 12.** Total ion chromatogram of volatiles collected from headspace of pure agar medium on day 10 (control).
