# Supplementary material for: Semiochemicals produced by fungal bark beetle symbiont Endoconidiophora rufipennis and the discovery of an anti-attractant for Ips typographus
Source: PLoS One. 2023 Apr 6;18(4):e0283906. doi: 10.1371/journal.pone.0283906 (PMC10079057; doi:10.1371/journal.pone.0283906)
Supplement: S1 Fig — (DOCX) [file pone.0283906.s004.docx]

## S3. Release rate dispensers
