# Supplementary material for: Semiochemicals produced by fungal bark beetle symbiont Endoconidiophora rufipennis and the discovery of an anti-attractant for Ips typographus
Source: PLoS One. 2023 Apr 6;18(4):e0283906. doi: 10.1371/journal.pone.0283906 (PMC10079057; doi:10.1371/journal.pone.0283906)
Supplement: Attachment — Submitted filename: Response to reviewers 230308.docx [file pone.0283906.s005.docx]

Response to Reviewers

Dear PlosONE, reviewers and Christopher Walton,

Thank you for your valuable comments, we did the following changes in the manuscript: We have now changed the formatting throughout the manuscript to ensure that the manuscript meets PLOS ONE's style requirements, including those for file naming. We have lifted the amended Funding Statement from the manuscript to our cover letter. Thank you for changing the online submission form. Minor edits are now changed throughout the manuscript as follow. Line number is referring to the document called “revised manuscript with track changes”.

**Following minor edits from reviewer #1 has been changed:**

Line 269: Write out ANOVA when first used. **Changes can be found on Line 278.**

Line 379: Missing full stop (period). **Changes can be found on Line 422.**

**Following minor edits from reviewer #2 has been changed:**

Lines 204-214 - indicate which compounds were provided by which chemical supplier. **Changes can be found on Line 201-209**

Line 222. I suggest adding a brief explanation of how the 3 compounds were selected for field testing in the methods – and not just in the discussion. After “Three chemicals were” insert “selected for testing in the field based on published reports of their activity in bark beetles (see Discussion for detailed rationale). The chemicals were”…. **Changes can be found on Line 222-224.**

Line 232. Change “For each dispenser, 1 mL of fungal compound was distributed onto the felt in the dispenser bag” to “For each of the test fungal compounds, 1 mL of synthetic pure compound was distributed onto the felt in a dispenser bag” **Changes can be found on Line 218-220.**

Line 243. For the Lindgren funnel traps, how many funnel units did they have? What killing agent was used in the collection cups – dry cups with insecticide strip, or wet cups with liquid preservative?

**Clarification can be found on Line 251-260, no killing agent was utilized in the field experiment. But we are aware that this may affect the results (usually lower catches due to that insects may escape or predadors may enter and the trap and kill/eat the caught spruce bark beetles).**

Line 389. What species were odour receptor neurons found in for 2-Phenethyl acetate? Is Ips typographus known to have receptors for this compound? **Clarification can be found on Line 431.**

Line 396. Add a reference for the statement that geranyl acetone has been reported as a common green leaf volatile **Clarification can be found on Line 439.**

Line 398. Add a reference for the statement that geranyl acetate has been demonstrated as an attractant or antiattractant in several taxa. **Assuming that the reviewer mean geranyl actone clarification can be found on Line 441-444 and refs added in Line 444.**

Line 404. Add a reference for the statement that geranyl acetate as EAD activity in Ips subelongatus. **Assuming that the reviewer mean geranyl actone as he/she refer to line 404 in the geranyl acetone paragraph. Reference added on Line 447.**

Line 410-411. Reword sentence – it seems awkward. “but electophsioloical activity alone does not 2-alone does not infer behavioral activity (attraction, inhibition, or none)” **Changes can be found on Line 451-3.**

Line 420. Summarize and cite any literature of known physiological or behavioral reponses to geranyl acetate. **Reference inserted on Line 462-3, geranyl acetate** **is a very common floral volatile and a very common semiochemical in diptera and Hymenoptera.**

Line 440. “have been reported as electophysiologically active” in what species? **Clarification can be found on Line 482.**

Line 467. Add a reference for the statement that Geranyl acetone is relatively low cost compound compared to other anti attractants, and for the anti-attraction activity of 1-octen-3-ol, trans-conophthorin, and thujanol. **Clarification can be found on Line 522-527. The price for thujanol is 250 USD for 5 gram and tr-conophthorin can only be bought from a small company**
<https://chemtronica.com>*.* ).

Line 468. Add a reference for the lower volatility of geranyl acetone compared to 1,8-cineole. **Reference can be found on Line 522-527.**

Line 477-478. Add a reference for the limited availability and high cost of other anti-attractants trans-conophthorin and verbenone.

**Clarification can be found on Line 532-536 trans-conophthorin can only be bought from a small company (**[**https://chemtronica.com**](https://chemtronica.com)**. ). and verbenone is used a lot in US for the mountain pine beetle so the supply of this natural product is depleted everywhere.**

Table 1. In addition to the relative amounts, it might be useful to give the total daily quantity of volatiles emitted. Although there is no quantitative internal standard when using SPME fibers for volatile collection, since the volume of the airspace was the same each day, and the length of time that the SPME fiber was exposed in the airspace, it seems that there should be a way to standardize the total amount of volatiles captured on each day and report them as ng or pg per mL of headspace or something like that? How do the volatile rates compare to the release rates of lures?

**The purpose of Table 1 is to indicate how the relative emitted proportions of the compounds change over time. Nothing else, so no change.**

Table 2. List the chemical supplier for each compound – perhaps as a footnote

**Not done, we believe it is enough to give the specified suppliers in the M&M section**

Figure 5. I suggest adding “+ Synth Pher” to each of the treatment labels on the X-axis to clarify and emphasize that each compound was tested in combination with the synthetic pheromone. I also suggest using the letter “a” on the three bars with the highest values and the letter “b” on the bar for Geranyl acetone with the lowest value. The insert with the schematic for the block layout is not necessary and kind of confusing.

**Removing an inset too small is a good suggestion, thanks!**

**Logically, the different treatment odours are added (“+”) to the control (pheromone), not the other way around. Adding 4 times the present longer acronym to the x-axis labels adds visual noise (overcrowding). Thus, a shorter acronym is used “Ph.” (synthetic is not vital as only 1 type of pheromone is used).**

**“a” is normally used before “b” from the alphabetic order inherited from the Phoenicians.**

**The changed fig. 5 can be found on Line 368-69**
